# A non-enzymatic glucose sensor based on electrospun 3-D copper oxide micro-nanofiber network films using carboxylic-functionalized poly(arylene ether ketone)s as templates†

**DOI:** 10.1039/c8ra09749f

**Published:** 2019-02-25

**Authors:** Mengzhu Liu, Yongpeng Wang, Haibo Zhang, Zhenhua Jiang

**Affiliations:** College of Materials Science and Engineering, Jilin Institute of Chemical Technology Jilin 132022 People’s Republic of China wyp4889@163.com; National and Local Joint Engineering Laboratory for Synthesis Technology of High Performance Polymer, Jilin University Changchun People’s Republic of China

## Abstract

Benefitting from the carboxylic functional group, the high performance polymer PCA-PAEK was first used as a template to produce 3-D rope-like CuO micro-nanofiber (CuO-MNF) network films *via* electrospinning and subsequent calcination. FT-IR proved the ion exchange reaction between the template and Cu^2+^ ions, and demonstrated the final structure of CuO when combined with EDX and XRD spectra. SEM and TGA revealed the small amount of Cu^2+^ immobilized on the template, resulting in small diameter (348 nm), short length and 3-D network structure of the CuO-MNFs. The CuO-MNFs were then investigated in detail for direct electrocatalytic oxidation of glucose, which was evaluated using cyclic voltammetry and chronoamperometry. Results revealed a higher sensitivity, faster response and better anti-interference than CuO-MNFs produced from traditional templates at +0.40 V. The improved performance was ascribed to the high surface-to-volume ratio and the excellent 3-D network structure after immobilization. Therefore, it was concluded that the functional group on PCA-PAEK determined the morphology and performance of the CuO-MNFs.

## Abstract 2

3-D network films of rope-like CuO-MNFs with high surface-to-volume ratio were prepared. The product is a promising electrode material for fabrication of amperometric enzymeless glucose sensors.

## Graphical abstract

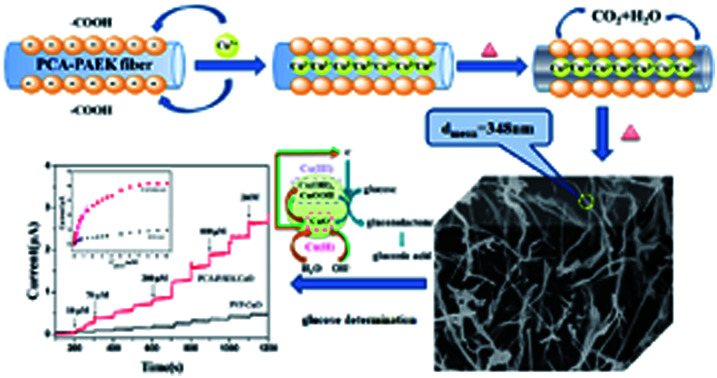

## Introduction

As a very important p-type semiconductor, copper oxide (CuO) has a narrow band gap of 1.2 eV. It has been extensively utilized in the fields of gas sensors, photochemical cells, catalysts and other electronic devices.^1^ Due to the wide application, many methods such as wet chemical methods, sonochemical methods, templating methods, as well as thermal decomposition methods,^2^ have been explored for the synthesis of CuO nanowires to improve their performance. But these methods either need complex precursor materials or experimental processes take a long time. Thus, other simple methods are required. Electrospinning is a straightforward, versatile and cost-effective top-down technique,^3–5^ which is often employed to fabricate one dimensional (1-D) micro-nanofibers.^6^ Electrospun micro-nanofibers possess properties of high porosity, high surface area-to-volume and length-to-diameter ratios, ease of modification and so on. Generally, the surface area of micro-nanofibers prepared through electrospinning is approximately double that of continuous thin films.^7^ The integration of these advantages makes micro-nanofibers have higher performance than other morphologies. Preparing metal oxides into nanofibers can combine their unique crystalline structures with large specific surface areas, which leads to excellent properties.^3^ Therefore, for CuO, if it was made into micro-nanofibers, the combination of electrochemical properties with the characteristics of nanofibrous materials will lead to the development of materials with outstanding electrochemical behavior.

To prepare metal oxide micro-nanofibers through electrospinning, polymer templates are essential. In recent reports,^8–12^ a variety of polymers such as poly(l-lysine), poly(vinyl pyrrolidone) (PVP), poly(vinyl acetate) (PVAc), polylactic acid (PLA), glycerin, pentaerythritol, *etc.* have been used as the templates to prepare nanoscale materials. Among these polymers, PVP as a nonionic hydrophilic polymer is the most commonly used template.^13–16^ However, it always results in extremely smooth nanofiber surfaces and excellent 1-D nanofiber structures, which are not beneficial for enhancing the properties.^17^ Therefore, researchers have paid attention to discovering a new template through which they can obtain a special structure to enhance the surface area.

**Chart 1:**
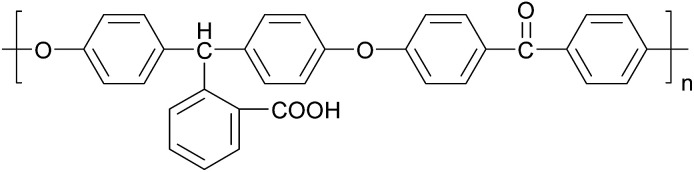
Structure of PCA-PAEK. PCA-PAEK: carboxylic acid modified poly(arylene ether ketone).

Poly(arylene ether ketone)s (PAEKs) are a kind of high performance polymer^18^ that possess excellent chemical resistance, high glass transition temperature, mechanical toughness, electrical properties and thermo-oxidative stability.^19^ Due to their high performance, PAEKs are widely used in advanced fields like aerospace,^20^ optical devices,^21^ and fuel cells.^22,23^ However, just because of the property of chemical resistance, PAEKs generally have poor solubility in a great variety of organic solvents,^24^ which severely restrict their application. Thus their functionalization has attracted significant attention.^25–27^ Carboxylic-functionalized PAEK (PCA-PAEK) (Chart 1) is a modified PAEK that introduces a carboxyl group on the polymer backbone. Introduction of –COOH can improve the solubility and chemical activity of PAEK. In our previous work,^28^ PCA-PAEK was fabricated into ultrafine fibers and the optimum condition was confirmed. However, as far as we know, it has never been used as a template to prepare CuO micro-nanofibers. The functional group (–COOH) on the polymer immobilizes the inorganic ions onto the polymer fibers, which enables the uniformity and thinness of the metal oxide fibers. Moreover, the morphology of the final metal oxide micro-nanofibers can be controlled by the amount of metal ions adsorbed on the polymer. Thus, PCA-PAEK can be utilized as a dispersant to prepare three dimensional (3-D) network films of CuO micro-nanofibers (CuO-MNFs) relying on the limited reaction between the functional group and copper ions. The functional group makes it possible for PCA-PAEK to act as a template to prepare metal oxide micro-nanofibers *via* electrospinning.

Herein, we report a new way of preparing CuO-MNFs using a new template by a combination of electrospinning with subsequent thermal treatment. PCA-PAEK was utilized as the template and dispersant to prepare 3-D network films of CuO-MNFs, which were applied for designing electrochemical enzymeless glucose sensors. The samples were elucidated using TGA, FT-IR, XRD and SEM. The formation mechanisms of the different structures were investigated. The potential application to glucose electro-oxidation of the activity of the prepared CuO-MNFs film electrode and its sensing performance were evaluated using cyclic voltammetry (CV) and chronoamperometry (*I*–*t*). This work provides an experimental and simple theoretical basis for further development of bioelectrochemical nanodevices for glucose determination by designing and fabricating a new type of CuO-MNFs.

## Experimental

### Chemicals and reagents

Carboxylic-functionalized poly(arylene ether ketone)s (PCA-PAEK, *M*_n_ = 31 016 g mol^−1^) was kindly provided by the Engineering Research Center of High Performance Plastics, Jilin University. Cupric acetate monohydrate (Cu(CH_3_COO)_2_·H_2_O, A.R.) was obtained from Sinopharm Chemical Regent Co., Ltd, China. Dichloromethane (DCM, Tianjin Tiantai Fine Chemical Co., Ltd., China) and *N*,*N*-dimethylformamide (DMF, Xilong Chemical Co., Ltd., China) were used as solvents directly. Poly(vinyl pyrrolidone) (PVP, *M*_w_ = 1 300 000) and Nafion perfluorinated resin solution (20 wt% in lower aliphatic alcohols and water, containing 34% water) were purchased from Sigma-Aldrich. d-Glucose, ascorbic acid (AA), uric acid (UA) and ethanol were purchased from Beijing Chemical Plant (Beijing, China). Glucose solutions with various concentrations were prepared with redistilled water. Solutions for anti-interference testing were prepared by dissolving interfering species in glucose solutions at normal physiological levels. All of the above chemicals were of analytical grade and directly used without any other treatment.

### Preparation of CuO-MNFs

PCA-PAEK granules were dissolved in a DMF/DCM mixture (the volume ratio was 5/5) to prepare a 20 wt% PCA-PAEK solution. In order to obtain a clear and homogeneous solution, the mixture was vigorously stirred for at least 6 h at room temperature. When the solution was transparent, it was loaded into a syringe. It was ensured that the amount of the solution was less than 3/4 of the syringe to avoid unnecessary overflow. Then the syringe was connected to a high-voltage supply (DW-P303-5AC High Voltage (0–30 kV), Dongwen High-voltage Power Supply Company, China), which mainly provided an electric field force to stretch the polymer into micro-nanofibers. At the other side, a collector placed 15 cm away from the orifice was connected to a grounded counter electrode. Then the applied voltage was adjusted to 17 kV, and at the same time a dense web of fibers could be collected on the aluminium foil. All electrospinning processes were carried out at ambient temperature. After the solution was consumed, in order to promote further evaporation of the solvent, the fibers were exposed to air overnight.

For the preparation of the CuO-MNFs, 6 wt% cupric acetate monohydrate solution was prepared beforehand. The completely dried PCA-PAEK pure polymer micro-nanofibers were then impregnated with cupric acetate solution for 10 h at 30 °C under continuous shaking. After the polymer micro-nanofibers were stained evenly, they could be taken out. Then the fiber membranes were washed with water three times and dried in a vacuum oven for 48 h at 45 °C to fully remove the unreacted compounds and solvent. The obtained PCA-PAEK/Cu(CH_3_COO)_2_ composite micro-nanofibers were calcined at 600 °C for 4 h in air subsequently.

In order to investigate the function of the PCA-PAEK templates, the most commonly used template, PVP, was also used to prepare CuO-MNFs. A transparent spinning solution containing 6 wt% cupric acetate was prepared by adding cupric acetate to 8 wt% PVP in ethanol, followed by magnetic stirring at ambient temperature for 6 h. The subsequent electrospinning and calcination process was the same as above.

The CuO-MNFs made from PCA-PAEK and PVP templates were respectively abbreviated as PAEK-CuO-MNFs and PVP-CuO-MNFs.

### Preparation of CuO-MNFs modified electrodes

A glassy carbon electrode (GCE) with diameter of 3 mm was polished with alumina slurries (1 mm and 0.05 mm sequentially) and then sonicated in deionized water, acetone, and deionized water successively and dried at room temperature. At this point, the electrode was ready for modification. Prior to the modification, a CuO-MNFs suspension was prepared. 5 mg of CuO-MNFs was added into 1 mL of ethanol, and then sonicated for 1 h to thoroughly disperse the micro-nanofibers. A 10 μL suspension was measured to drop onto the surface of the GCE. To immobilize CuO-MNFs onto the electrode surface, the solvent was evaporated under an infrared lamp. After the electrode was thoroughly dried, 5 μL of a 1 wt% Nafion solution was finally applied to serve both as a permselective membrane and entrapment matrix for the immobilization of CuO-MNFs.

### Characterization

A Japan SHIMADZU SSX-550 scanning electron microscope (SEM) equipped with an energy-dispersive X-ray spectrometer (EDX) was used to examine the morphology and composition of the as prepared samples. The image visualization software ImageJ was used to analyze the mean diameters of the composite micro-nanofibers. 100 measurements per field were chosen based on the SEM images.^29^ Thermal gravimetric analysis (TGA) was performed on a PerkinElmer Pyris 1 TGA (United States) from room temperature to 800 °C under a flowing air atmosphere. The vibration in the functional groups of the micro-nanofibers was analyzed using a Japan SHIMADZU 1.50SU1 Fourier transform-infrared radiation (FT-IR) spectrometer. X-ray powder diffractometry (XRD) was conducted on a Siemens X-ray diffractometer (D5005XRD) to study the crystal structure of the calcined micro-nanofibers. Scans were set from 30 to 70° (2*θ*). All electrochemical experiments were performed on a CHI 660E Electrochemical Workstation (CH Instruments, USA), using a traditional three-electrode electrochemical cell (a working volume of 5 mL) with a working electrode, a saturated calomel electrode, and a platinum wire counter electrode.

## Results and discussion

### Characterization of CuO-MNFs

**Fig. 1:**
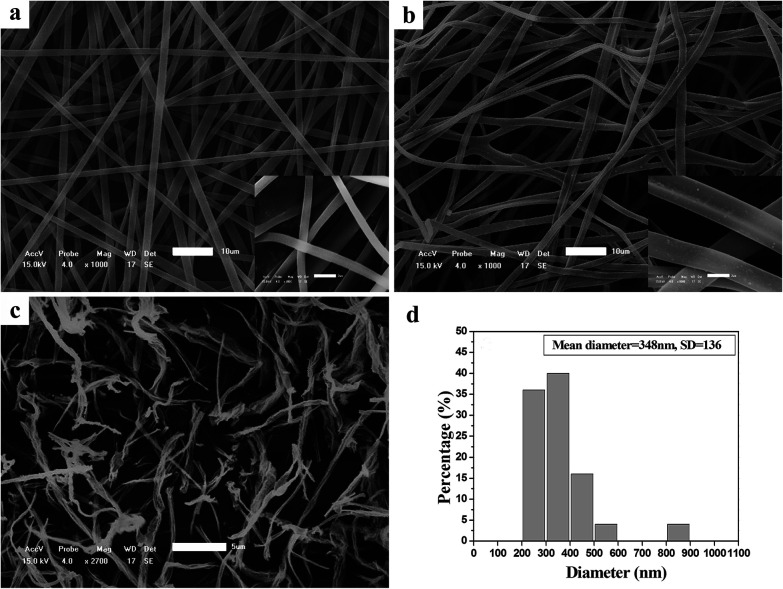
SEM images of (a) pure PCA-PAEK micro-nanofibers, and (b) PCA-PAEK/Cu(CH_3_COO)_2_ composite micro-nanofibers (the scale bar is 10 μm). Inset images are the corresponding higher magnifications. (c) PCA-PAEK/Cu(CH_3_COO)_2_ composite micro-nanofibers after calcination at 600 °C (the scale bar is 5 μm) and the corresponding fiber diameter distribution (d).

Fig. 1 illustrates SEM images of the as-spun micro-nanofibers. Excellent 1-D micro-nanofibers were observed. The length of the fibers could even reach centimeter grade. The fibers collected were aligned randomly because of the bending instability associated with the spinning jet. In Fig. 1(a), each individual pure PCA-PAEK micro-nanofiber was uniform in cross section. But the fiber after immersion became coarse, irregular, distorted, and intertwined at many loci. In addition, some fibers stuck together to form larger fibers. This was due to the long-time immersion which swelled the PCA-PAEK fibers. From the inset images, it can be seen that pure PCA-PAEK micro-nanofibers were smooth. However, after immersion in inorganic salt solution (Fig. 1(b)), the surface of the fibers was slightly modified. There were some dots. This was related to the reaction between Cu^2+^ and the carboxyl on PCA-PAEK. The particles dispersed throughout the fibers, which demonstrated that the PCA-PAEK polymer chain can act as a stabilizing agent to inhibit the agglomeration of the nanoparticles.^30^ The probable equation in the immersion process is shown in eqn (1).

1 Cu(CH_3_COO)_2_ + 2PAEK–COOH → (PAEK–COO)_2_Cu + 2CH_3_COOH

Cupric acetate and PCA-PAEK took part in an ion exchange reaction, due to the stronger metal coordination ability of –COOH on the PCA-PAEK. After being calcined at 600 °C for 3 hours, the excellent 1-D CuO micro-nanofibers were exfoliated to short nanofibers. Rope-like CuO-MNFs with mean diameter of 348 nm could be observed. They were not continuous fibers with length of a centimeter any more. The fibers were slim and short. They composed a uniform 3-D network-like structure, which may be related to the little amount of Cu^2+^ reacted with –COOH on the polymer. (SEM images of PVP-CUO-MNFS are provided in ESI Fig. S1.† It can be seen clearly that the morphology of PCA-PAEK-CUO-MNFS had a big difference to that of PVP-CUO-MNFS.)

**Fig. 2:**
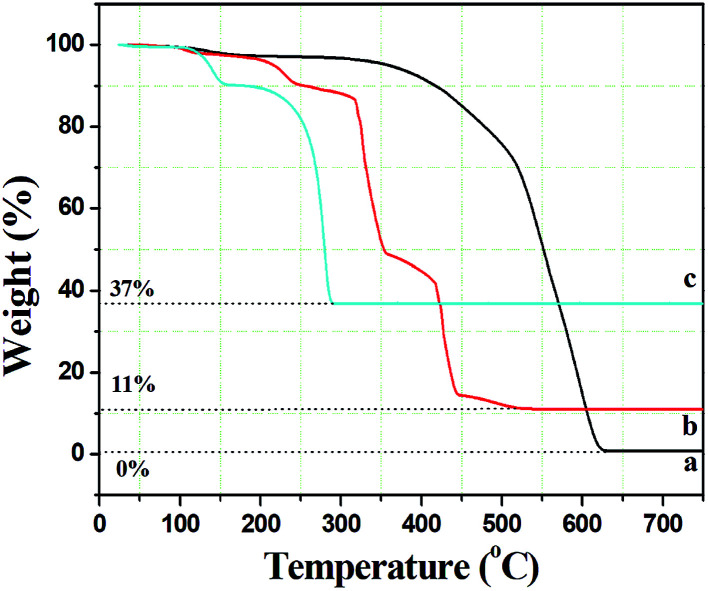
Thermal gravimetric analysis curves of (a) pure PCA-PAEK micro-nanofibers, (b) PCA-PAEK/Cu(CH_3_COO)_2_ composite micro-nanofibers, and (c) pure Cu(CH_3_COO)_2_·H_2_O.

In order to assess the appropriate calcination temperature and prove the speculation in the SEM analysis, the thermal behaviour of the composite fibers was investigated using thermal gravimetric analyses (TGA). As seen from Fig. 2(a), there was only one weight loss stage for pure PCA-PAEK fibers during 350 °C to 630 °C, which corresponded to the decomposition of the polymer main chain. The curve is very flat before 350 °C, which demonstrates the excellent thermo-oxidative stability of the polymers. After 630 °C, no more weight loss can be seen, indicating PCA-PAEK was decomposed completely. The TGA curve of pure Cu(CH_3_COO)_2_·H_2_O (Fig. 2(c)) powders exhibits two stages of decomposition: the first stage appears from 112 to 151 °C and is attributed to partial decomposition of Cu(CH_3_COO)_2_·H_2_O and liberation of the crystal water; the second stage occurs in the range of 202–288 °C and is related to the complete decomposition of Cu(CH_3_COO)_2_. Based on the analyses above, the thermal behavior of PCA-PAEK/Cu(CH_3_COO)_2_ can be easily found (shown in Fig. 2(b)). The slight weight loss (∼10%) from room temperature to 239 °C corresponds to the loss of moisture. The first dramatic weight loss (∼45%) occurs at 315–354 °C and is attributed to decomposition of Cu(PAEK-COO)_2_ and the PCA-PAEK side chain. The second dramatic weight loss (∼35%) occurs at 418–443 °C and is due to decomposition of the PCA-PAEK main chain. The shift of the decomposition temperature of Cu(CH_3_COO)_2_ and PCA-PAEK should be due to the reaction between the –COOH on the polymer chains and the metal ions. In addition, the existence of inorganic salt can also facilitate the decomposition of the polymer. When the temperature reaches about 500 °C, the curve becomes flat, demonstrating that the composite micro-nanofibers had transformed into inorganic oxide completely. Thus, the calcined temperature was determined at 600 °C. In general, the following reaction took place during the calcination process and led to the formation of metal oxide:

2 (PAEK–COO)_2_Cu → CuO + H_2_O + CO_2_

Moreover, the percentage of the residue was only about 11%, indicating that the amount of Cu^2+^ absorbed on the polymer side chain was small, which proved the speculation in the SEM analysis.

**Fig. 3:**
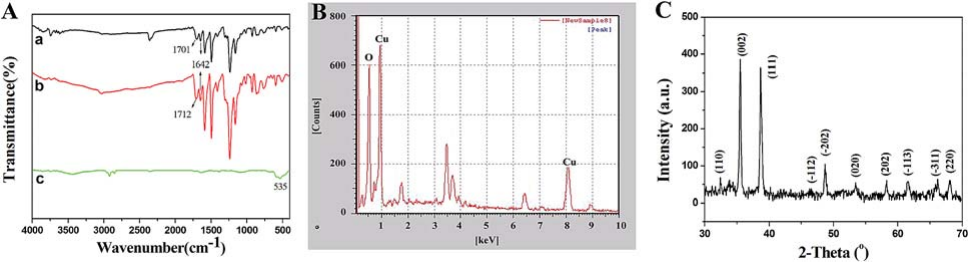
(A) FT-IR spectra of (a) pure PCA-PAEK micro-nanofibers, PCA-PAEK/Cu(CH_3_COO)_2_ composite micro-nanofibers (b) before and (c) after calcination at high temperature; (B) and (C) are EDX and XRD spectra of the calcined PCA-PAEK/Cu(CH_3_COO)_2_ composite micro-nanofibers, respectively.

The structure of the product calcined at high temperature was identified using FT-IR, XRD and EDX. FT-IR was investigated in order to demonstrate the presence of intermediate products and identify the structure of the final products preliminarily. As shown in Fig. 3A(a), the weak peak at 1642 cm^−1^ belongs to the stretching vibration of C=O on the main chain of PCA-PAEK. After mixing with metal salt, the peak did not shift (Fig. 3A(b)). The peak at 1701 cm^−1^ in Fig. 3A(a) corresponds to the asymmetric stretching vibration of C=O on the side chain of pure PCA-PAEK.^31^ However, it shifts to 1712 cm^−1^ in Fig. 3A(b). This demonstrates that the –COOH on the side chain of PCA-PAEK had reacted with metal ions already, which agrees with the speculation in the SEM analysis. After treating at 600 °C, the organic groups disappeared and new peaks appear. In Fig. 3A(c), the peak at about 535 cm^−1^ is the stretching vibration of Cu–O,^32^ demonstrating the production of pure CuO.

From Fig. 3(B), it can be seen clearly that there were O and Cu elements in the calcined PCA-PAEK/Cu(CH_3_COO)_2_ composite micro-nanofibers, demonstrating the presence of CuO products. Besides, other elements like Fe, Sn and Si were also present. This may be related to the chemical constituents of the raw material. Table 1 gives quantity analysis of every element in Fig. 3(B). It can be seen that the atomic percentage of CuO nearly reaches 90%, whereas impurities are less than 10%. Thus, impurities can be ignored.

**Table 1:** Quantity analysis of the elements in Fig. 3(B)

Sample	Element	Intensity	AT%
CuO micro-nanofiber	O	3.284	68.585
	Cu	1.009	21.338
	Fe	0.347	3.262
	Si	0.514	1.899
	Sn	1.435	4.916

XRD spectra of the calcined samples are shown in Fig. 3(C). After calcination at high temperature, the micro-nanofibers exhibited well defined diffraction peaks, indicating that the products were perfectly crystallized. Among these diffraction peaks, the highest peaks located at 2*θ* = 35.5° and 38.7°, indexed as the (111)–(002) and (111)–(200) planes, respectively, were the characteristics for the pure phase monoclinic CuO crystallites. All of the other diffraction peaks can also be assigned to monoclinic structured CuO (Joint Committee for Powder Diffraction Studies (JCPDS) file no. 05-0661).^33^ XRD analysis demonstrated once again that the structure of the product was CuO.

**Fig. 4:**
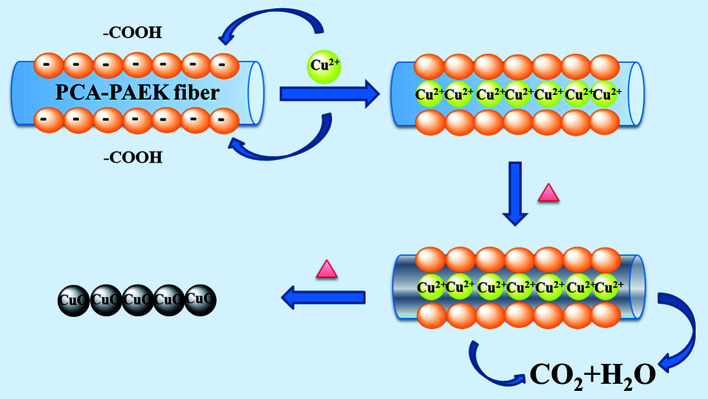
The possible formation mechanism of CuO-MNFs using PCA-PAEK as the template.

Referring to the analysis above, the possible mechanism to prepare CuO-MNFs by using PCA-PAEK as the template was speculated and is shown in Fig. 4. Firstly, when pure PCA-PAEK with COOH on the side chains was immersed in Cu^2+^ solution, the metal ions reacted with COO– and were immobilized on the fibers. With the increase in temperature, the polymer templates decomposed and the metal ions were oxidized into metal oxides gradually. At the same time, due to the good stability of PAEK, the fiber morphology was retained. Therefore pure CuO-MNFs were obtained. However, due to the small amount of metal ions adsorbed on the PCA-PAEK fibers, when the templates decomposed thoroughly, the long micro-nanofibers were exfoliated to short micro-nanofibers, accompanying the transformation from 1-D micro-nanofibers to a 3-D network structure. It was easy to find out that in the formation of the CuO-MNFs with a special morphology, the ion exchange reaction between metal acetate and PCA-PAEK was the key point.

### Sensitive and selective glucose sensing with CuO-MNFs modified electrode

**Fig. 5:**
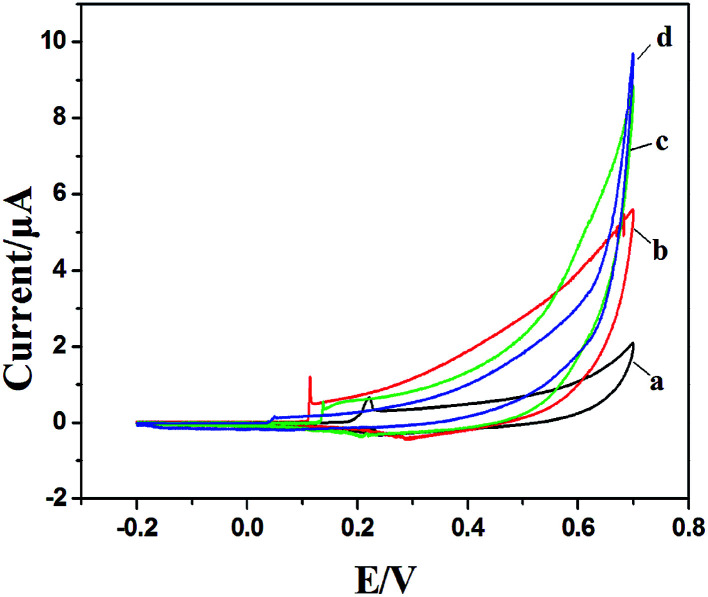
CV of PVP-CuO-MNFs-GCE and PCA-PAEK-CuO-MNFs-GCE in 0.1 M NaOH in the absence (a and c) and presence of 4 mM glucose (b and d) respectively at 50 mV s^−1^.

**Fig. 6:**
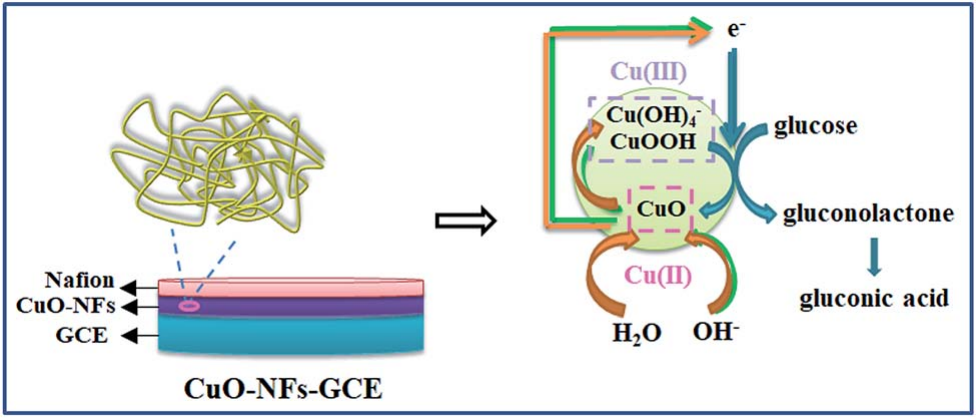
Schematic representation of CuO-MNFs-GCE and its glucose sensing mechanism.

PVP is a common polymer to prepare metal oxide micro-nanofibers. In order to establish the differences with the new template (PCA-PAEK), PVP-CuO-MNFs-GCE was used as the contrast experiment. Electrocatalytic activity of the CuO modified electrode towards oxidation of glucose was tested through CV, which was carried out in the potential range from 0 to +0.70 V in the absence and presence of glucose in 0.1 M NaOH. As shown in Fig. 5, a pair of redox peaks is observed for both PVP-CuO-MNFs-GCE and PCA-PAEK-CuO-MNFs-GCE, which can be assigned to the Cu(ii) and Cu(iii) redox couple. PVP-CuO-MNFs-GCE (curve a) shows a smaller background current in 0.1 M NaOH, while PCA-PAEK-CuO-MNFs-GCE (curve c) exhibits a dramatic increase in current signal toward the positive end of the potential range. The phenomenon was ascribed to the role of PCA-PAEK templates which had increased the electroactive surface area,^33^ leading to a high surface energy and enhanced electron transfer of the as-prepared CuO-MNFs. In the presence of 4 mM glucose, the background current and the anodic peak increased for both of the two electrodes, which can be attributed to the catalytic effect of the redox couple for oxidation of glucose to gluconic acid. The oxidation mechanism (shown in Fig. 6) was believed to take place *via* the reaction as shown in eqn (3)–(5).^34^

3 CuO + OH^−^ → CuOOH + e^−^ or CuO + H_2_O + 2OH^−^ → Cu(OH)_4_^−^ + e^−^

4 Cu(iii) + glucose + e^−^ → gluconolactone + Cu(ii)

5 Gluconolactone → gluconic acid

Firstly, CuO was electrochemically oxidized to strong oxidizing Cu(iii) species such as CuOOH or Cu(OH)_4_^−^. And then, glucose was catalytically oxidized by the Cu(iii) species to produce gluconic acid. In alkaline solution, Cu(iii) rapidly oxidized glucose to gluconolactone, and then gluconic acid. The consumption of Cu(iii) and production of Cu(ii) led to the presence of an oxidation peak and reduction peak. Herein, the Cu(iii) might act as an electron-transfer mediator. The reaction manifested the oxidation of glucose on CuO modified GCE in an alkaline solution as a nonenzymatic electro-oxidation process. The results indicated that the electrodes may have potential application in glucose detection.

**Fig. 7:**
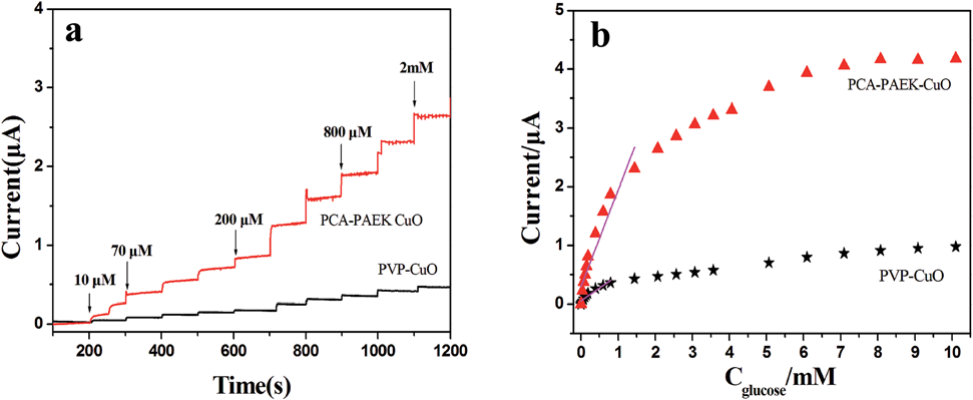
(a) Amperometric response of CuO-MNFs-GCE with successive additions of 0.1 mM glucose to 0.1 M NaOH at +0.40 V and (b) calibration curve for the amperometric responses of CuO-MNFs-GCE to glucose.

Amperometric analysis was carried out at +0.40 V for PCA-PAEK-CuO-MNFs-GCE and PVP-CuO-MNFs-GCE by successive addition of glucose to 0.1 M NaOH. From Fig. 7a, it can be seen that both of the two CuO-MNFs-GCEs responded rapidly to the change in glucose concentration. The time for achieving 95% of the steady-state current for PCA-PAEK-CuO-MNFs-GCE and PVP-CuO-MNFs-GCE was within 3 s and 5 s, respectively, indicating a better catalytic property of the PCA-PAEK-CuO-MNFs-GCE. Moreover, the current response of PCA-PAEK-CuO-MNFs-GCE to 2 mM glucose was approximately 2.65 μA, which was significantly higher than that of PVP-CuO-MNFs-GCE. Fig. 7b shows the corresponding calibration curve. A better linear response for the electrocatalytic current of glucose can be seen for the PCA-PAEK-CuO-MNFs-GCE.

As listed in Table 2, PCA-PAEK-CuO-MNFs-GCE exhibited higher sensitivity, a lower detection limit and a wider linear range than PVP-CuO-MNFs-GCE. The superior behaviour of PCA-PAEK-CuO-MNFs-GCE can be attributed to the unique structure of the 3-D CuO-MNFs network, which was formed by the 1-D CuO-MNFs and greatly enhances the specific surface area. This provides more chance to shuttle electrons between glucose and the working electrode.^33^ Therefore, the reaction of glucose oxidation which mainly relies on electron transfer (shown in Fig. 6) can be enhanced. From the analysis above, it can be found that using PCA-PAEK as the template to produce CuO-MNFs can provide a greater possibility for the formation of a 3-D CuO-MNFs network through the ion exchange reaction between the functional group on the polymers and metal ions, thereby increasing the specific surface area efficiently.

**Table 2:** Analytical characteristics of PVP-CuO-MNFs-GCE and PCA-PAEK-CuO-NFs-GCE at an applied potential of +0.4 V

	PVP-CuO-MNFs/GCE	PCA-PAEK-CuO-MNFs/GCE
Linear range up to/mM	0.78	1.43
Detection limit/μM	65.3	7.07
Sensitivity/μA mM^−1^cm^−2^	6.17	23.35

**Fig. 8:**
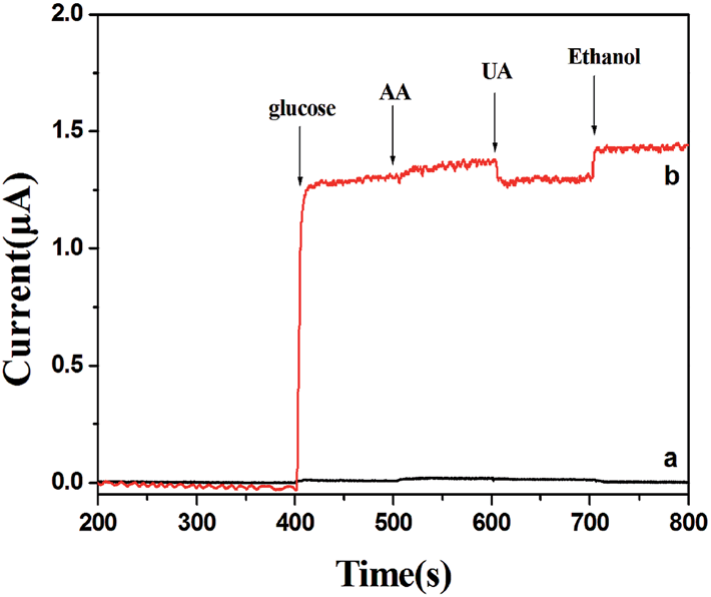
Amperometric responses of (a) PVP-CuO-MNFs-GCE and (b) PCA-PAEK-CuO-MNFs-GCE with successive additions of 3 mM glucose, 0.1 mM AA, 0.1 mM UA, and 10 mM ethanol to 0.1 M NaOH at +0.40 V.

Good anti-interference is a very important but challenging aspect for a non-enzymatic glucose sensor. This is because the co-existence of electroactive compounds in real blood (such as AA and UA) interferes with the determination of glucose. It is well known that the common concentration of glucose in blood is 3–8 mM and those of the interfering species are about 0.1 mM. Therefore, we examined the electrochemical responses for the possible interfering species such as UA, AA and ethanol. The experiment was carried out by adding 3 mM glucose in 0.1 M NaOH followed by 0.1 mM AA, 0.1 mM UA and 10 mM ethanol. The results are shown in Fig. 8. For PVP-CuO-MNFs-GCE (Fig. 8(a)), the signal was not obvious, indicating a poor response and distinguishability. While for the PCA-PAEK-CuO-MNFs-GCE (Fig. 8(b)), the signal was evident. UA caused insignificant interference at a physiological level, and AA and ethanol did not cause any observable interference. The results implied that the activated PCA-PAEK-CuO-MNFs-GCE has good anti-interference ability. Differences can be found between PVP-CuO-MNFs-GCE and PCA-PAEK-CuO-MNFs-GCE when they had a response to UA. A repelling model could be applied to explain the difference. It has been reported^35^ that the negatively charged surfaces of micro-nanofibers in strong alkaline solution can repel deprotonated UA, thus reducing the electro-oxidation of UA. Due to the ion exchange reaction between PCA-PAEK and Cu^2+^, 3-D micro-nanofibers formed so as to increase the electroactive surface area. Thus, this provided a greater possibility to repel deprotonated UA, resulting in an evident response.

In a word, PCA-PAEK-CuO-MNFs-GCE exhibited better performance such as anti-interference, sensitivity and electro-oxidation of glucose. The reason for these phenomena was the enlarged surface area and 3-D network structure of the CuO-MNFs, which was produced by the ion exchange reaction between the active –COOH on the PCA-PAEK template and metal ions.

## Conclusions

3-D network films of rope-like CuO-MNFs with high surface-to-volume ratio were prepared through electrospinning using PCA-PAEK as templates, which had active –COOH on the side chain and led to the adsorption of metal ions. SEM and TGA indicated that the amount of Cu^2+^ adsorbed on the template micro-nanofibers was very small, resulting in small diameter, short length and a 3-D network structure of the final CuO-MNFs. FT-IR proved that the template had reacted with the metal ions and demonstrated that the products were highly pure CuO when combined with EDX and XRD spectra. The formation mechanism of the rope-like CuO-MNFs was speculated. It showed that the morphology was determined by the ion exchange reaction between the metal acetate and PCA-PAEK. We demonstrated the possibility of the electrospun CuO-MNFs’ application for direct glucose determination. Compared with electrodes modified with CuO-MNFs made from traditional polymer templates, the PCA-PAEK-CuO-MNFs modified electrodes exhibited superiorities of good anti-interference, high sensitivity and fast response to glucose, attributed to the large surface area and 3-D network structure, which benefited from the ion exchange reaction between the template and metal ions. In a word, PCA-PAEK was first used as a template to prepare CuO-MNFs with a special morphology through a simple chemical reaction. The functional group on the polymer side chain determined the morphology and performance of the final product. The product is a promising electrode material for fabrication of amperometric enzymeless glucose sensors.

## Conflicts of interest

There are no conflicts to declare.

## Supplementary Material

RA-009-C8RA09749F-s001

## References

[cit1] Sahay R., Kumar P. S., Aravindan V., Sundaramurthy J., Ling W. C., Mhaisalkar S. G., Ramakrishna S., Madhavi S. (2012). J. Phys. Chem. C.

[cit2] Wu H., Lin D., Pan W. (2006). Appl. Phys. Lett..

[cit3] Savva I., Kalogirou A. S., Chatzinicolaou A., Papaphilippou P., Pantelidou A., Vasile E., Vasile E., Koutentis P. A., Christoforou T. K. (2014). RSC Adv..

[cit4] Deshawar D., Chokshi P. (2017). Polymer.

[cit5] Wittmer C. R., Hebraud A., Nedjari S., Schlatter G. (2014). Polymer.

[cit6] Dai Y., Liu W., Formo E., Sun Y., Xia Y. (2011). Polym. Adv. Technol..

[cit7] Dersch R., Steinhart M., Boudriot U., Greiner A., Wendorff J. H. (2005). Polym. Adv. Technol..

[cit8] Sarah L. S., David W. W. (2006). Chem. Mater..

[cit9] Nuansing W. W., Ninmuang S., Jarernboon W., Maensiria S., Seraphin S. (2006). Mater. Sci. Eng., B.

[cit10] Ding B., Kim C. K., Kim H. Y., Seo M. K., Park S. J. (2004). Fibers Polym..

[cit11] Zheng J. Y., Pang J. B., Qiu K. Y., Wei Y. (2001). J. Mater. Chem..

[cit12] Zheng J. Y., Qiu K. Y., Feng Q. W., Xu J. G., Wei Y. (2000). Mol. Cryst. Liq. Cryst..

[cit13] Santibenchakul S., Chaiyasith S., Pecharap W. (2016). Integr. Ferroelectr..

[cit14] Caratão B., Carneiro E., Sá P., Almeida B., Carvalho S. (2014). J. Nanotechnol..

[cit15] Khan W. S., Asmatulu R., Lin Y. H., Chen Y. Y., Ho J. C. (2012). J. Nanotechnol..

[cit16] Elayappan V., Panneerselvam P., Nemala S., Nallathambi K. S., Angaiah S. (2015). Appl. Phys. A: Mater. Sci. Process..

[cit17] Liu M., Cheng Z., Yan J., Qiang L., Ru X., Liu F., Ding D., Li J. (2013). J. Appl. Polym. Sci..

[cit18] Wang F., Hickner M., Kim Y. S., Zawodzinski T. A., McGrath J. E. (2002). J. Membr. Sci..

[cit19] Wang F., Chen T., Xu J., Liu T., Jiang H., Qi Y., Liu S., Li X. (2006). Polymer.

[cit20] Sayyed M. M., Maldar N. N. (2010). Mater. Sci. Eng., B.

[cit21] Qi Y., Ding J., Day M., Jiang J., Callender C. L. (2005). Chem. Mater..

[cit22] Shang X., Li X., Xiao M., Meng Y. (2006). Polymer.

[cit23] Shang X., Tian S., Kong L., Meng Y. (2005). J. Membr. Sci..

[cit24] Ohno M., Takata T., Endo T. (1994). Macromolecules.

[cit25] Baek J. B., Qin H., Mather P. T., Tan L. S. (2002). Macromolecules.

[cit26] Guo M., Liu B., Guan S., Liu C., Qin H., Jiang Z. (2010). J. Membr. Sci..

[cit27] Geng Z., Ba J., Zhang S., Luan J., Jiang X., Huo P., Wang G. (2012). J. Mater. Chem..

[cit28] Liu M., Wang Y., Cheng Z., Zhang M., Hu M., Li J. (2015). High Perform. Polym..

[cit29] Liu M., Wang Y., Cheng Z., Zhang M., Hu M., Li J. (2014). Appl. Surf. Sci..

[cit30] Liu M., Song L., Wang Y., Cheng Z., Li J. (2014). High Perform. Polym..

[cit31] Zhang Y. (2016). High Perform. Polym..

[cit32] Guan H., Shao C., Chen B., Jian G., Yang X. (2003). Inorg. Chem. Commun..

[cit33] Wang W., Zhang L., Tong S., Li X., Song W. (2009). Biosens. Bioelectron..

[cit34] Huang F., Zhong Y., Chen J., Li S., Li Y., Wang F., Feng S. (2013). Anal. Methods.

[cit35] Ding Y., Wang Y., Zhang L. C., Zhang H., Lei Y. (2012). J. Mater. Chem..

