# Supplementary material for: A non-enzymatic glucose sensor based on electrospun 3-D copper oxide micro-nanofiber network films using carboxylic-functionalized poly(arylene ether ketone)s as templates
Source: RSC Adv. 2019 Feb 25;9(12):6613–9. doi: 10.1039/c8ra09749f (PMC9060950; doi:10.1039/c8ra09749f)
Supplement: RA-009-C8RA09749F-s001 [file RA-009-C8RA09749F-s001.pdf]

Electronic Supplementary Material (ESI) for RSC Advances.
This journal is © The Royal Society of Chemistry 2019

Supporting Information

Morphologies of PVP/Cu(CH_3COO)$_2$ composite micro-nanofibers before and after calcination were given in Fig.S1. It can be observed that the as-spun composite micro-nanofibers appeared relatively smooth and round. They lay randomly, crossed and overlapped with each other, forming lots of distinct junctions. At each of these junctions, the upper fiber was slightly suspended due to the presence of the lower one. After calcination at high temperature, the morphology was modified seriously. The fibers shrunk and gathered closely to form a large cluster. The mean diameter was 1347nm, which was much bigger than that of PCA-PAEK-CuO-MNFs (348nm). Although there were some pores on the cluster, its specific area was apparently less than that of 3-D CuO-MNFs network made from PCA-PAEK/Cu(CH_3COO)$_2$ composite micro-nanofibers. This was the essential reason for PVP-CuO-MNFs-GCE to present worse sensing behaviors to glucose than PCA-PAEK-CuO-MNFs-GCE.

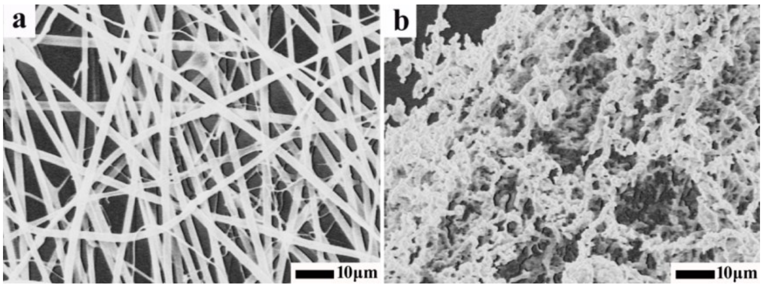

Fig.S1 SEM images of PVP/Cu(CH_3COO)$_2$ composite micro-nanofibers (a) before and (b) after calcination at 600 ℃.
